# 1143. Safety and Efficacy of Ceftolozane/Tazobactam Versus Meropenem in Neonatal and Pediatric Participants With Complicated Urinary Tract Infection, Including Pyelonephritis: A Phase 2, Randomized, Clinical Trial

**DOI:** 10.1093/ofid/ofab466.1336

**Published:** 2021-12-04

**Authors:** Emmanuel Roilides, Negar Ashouri, John S Bradley, Matthew G Johnson, Julia Lonchar, Feng-Hsiu Su, Jennifer A Huntington, Myra W Popejoy, Mekki Bensaci, Carisa S De Anda, Elizabeth G Rhee, Christopher Bruno

**Affiliations:** 1 Aristotle University and Hippokration General Hospital, Thessaloniki, Thessaloniki, Greece; 2 CHOC Children’s, Orange, California; 3 University of California San Diego, San Diego, California; 4 Merck & Co., Inc., Kenilworth, NJ

## Abstract

**Background:**

Ceftolozane/tazobactam (C/T) is a cephalosporin–β-lactamase inhibitor combination approved to treat complicated urinary tract infections (cUTI), complicated intra-abdominal infections, and nosocomial pneumonia in adults. Safety and efficacy of C/T in neonatal and pediatric participants with cUTI was assessed.

**Methods:**

This phase 2, randomized, double-blind study (NCT03230838) compared C/T with meropenem (MEM) for treatment of cUTI, including pyelonephritis in participants from birth to 18 years of age. Treatment duration was 7-14 days. After 3 days of intravenous therapy, optional oral step-down therapy was allowed. Participants were stratified and dosed by age group (Table 1). The primary objective was to evaluate the safety and tolerability of C/T compared with MEM, and key secondary end points included clinical response and per-participant microbiologic response at end of treatment (EOT) and test of cure (TOC).

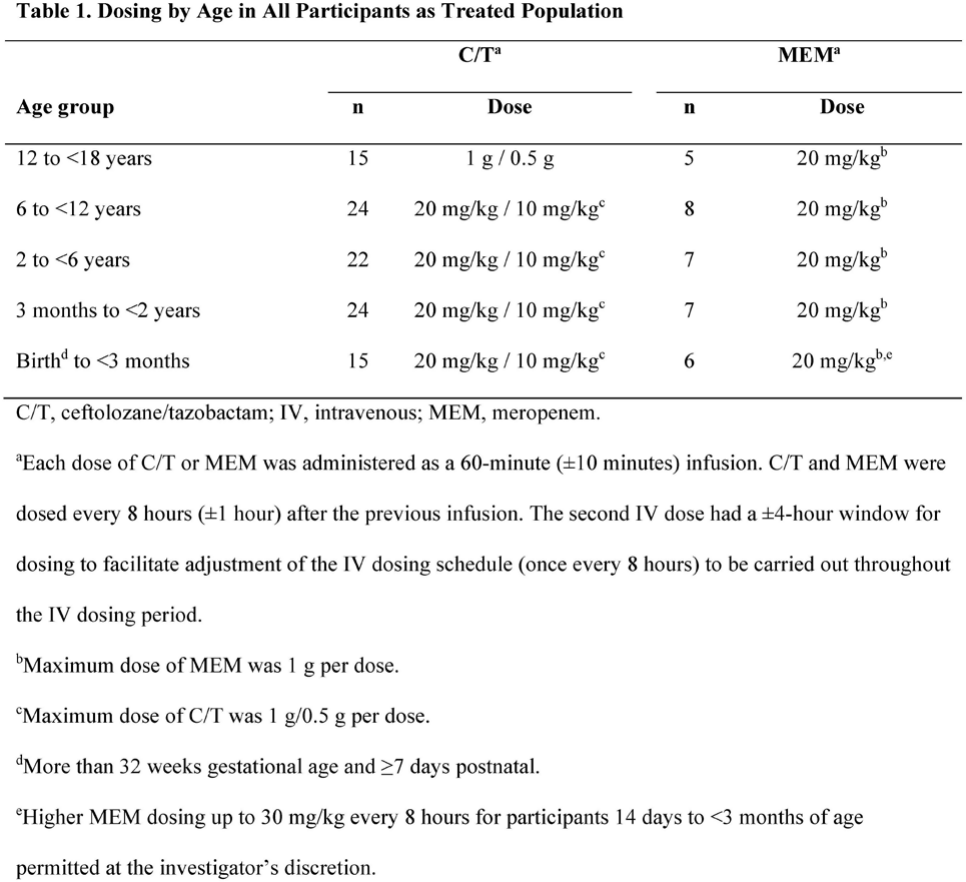

**Results:**

Participants were randomized 3:1 and treated with C/T (n=100) or MEM (n=33). The microbiologic modified intent-to-treat population (mMITT) included 95 participants in the C/T (n=71) and MEM (n=24) arms; the most common reason for mMITT exclusion was lack of a qualifying baseline uropathogen (28.4%). Pyelonephritis was the most common baseline diagnosis (83.2%), and Escherichia coli was the most common qualifying baseline uropathogen (77.9%). Overall mean treatment duration was comparable in both arms (C/T, 10.2 days; MEM, 10.7); a total of 50 (70.4%) and 20 (83.3%) participants switched to optional oral step-down therapy in the C/T and MEM arms, respectively, both for a mean of approximately 6 days. The overall incidence of adverse events (AE; all and drug related), serious AE (SAE), and AE leading to discontinuation was comparable between C/T and MEM arms. There were no AE leading to death, drug-related SAE, or discontinuations due to drug-related AE or SAE (Table 2). For C/T and MEM, rates of clinical cure and microbiologic eradication at EOT and TOC were high (Figure).

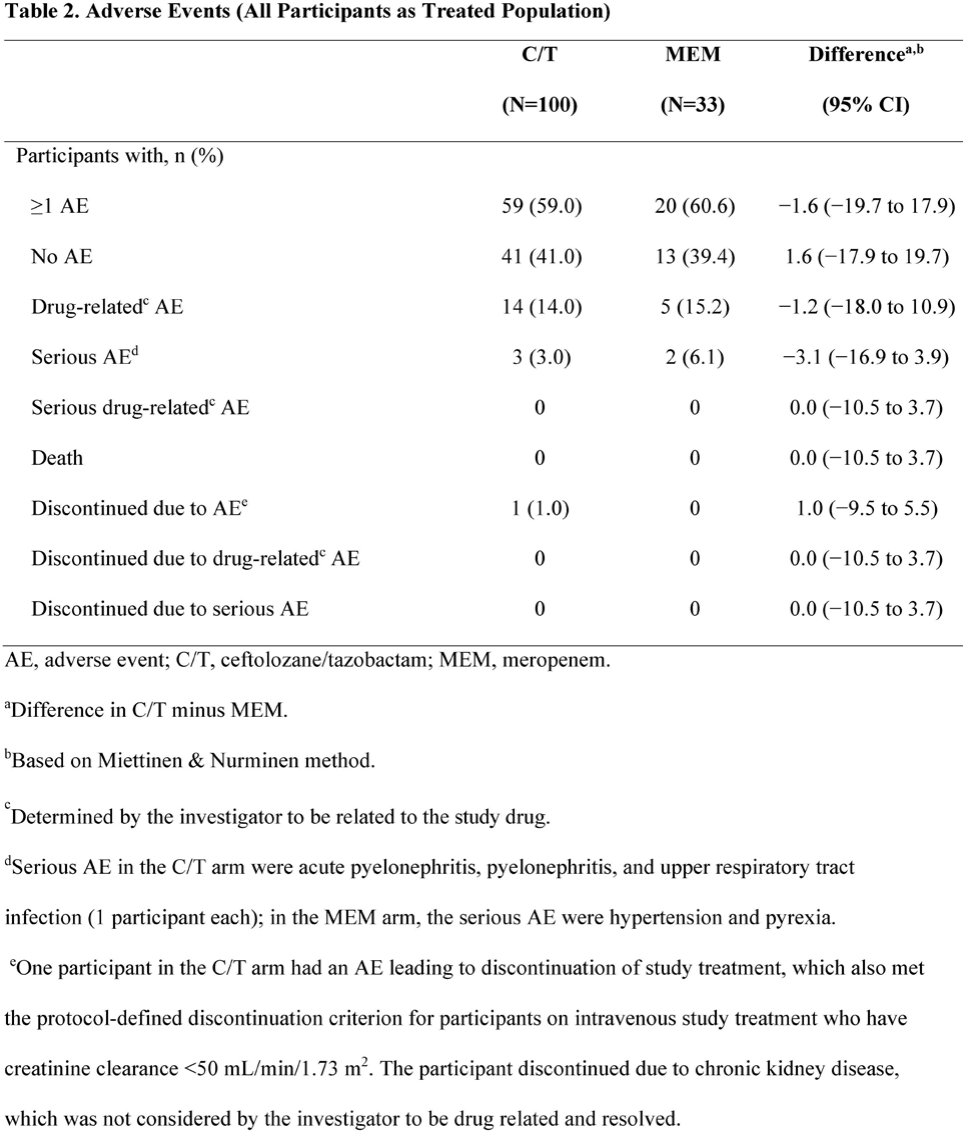

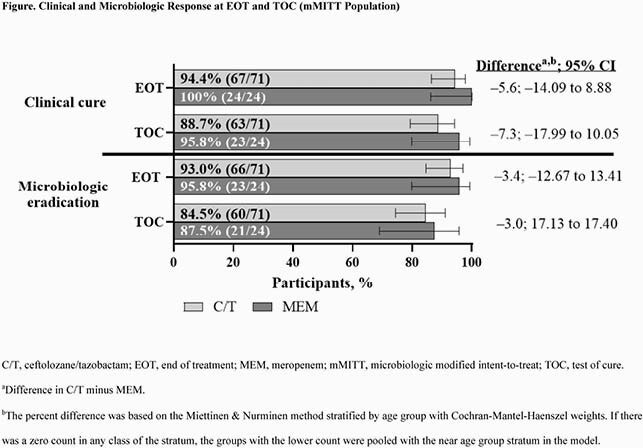

**Conclusion:**

In this study, C/T was well tolerated with a safety profile comparable to MEM and to the previously reported safety profile for C/T in adults with cUTI. C/T achieved high clinical cure and microbiologic eradication rates and is a potential new treatment option for children with cUTI.

**Disclosures:**

**Emmanuel Roilides, MD, PhD, FIDSA, FAAM, FESCMID**, **Merck Sharp & Dohme Corp.** (Consultant, Grant/Research Support) **Negar Ashouri, MD**, **Merck Sharp & Dohme Corp.** (Grant/Research Support) **Matthew G. Johnson, MD**, **Merck Sharp & Dohme Corp., a subsidiary of Merck & Co., Inc., Kenilworth, NJ, USA** (Employee) **Julia Lonchar, MSc**, **Merck Sharp & Dohme Corp.** (Employee, Shareholder) **Feng-Hsiu Su, MPH, MBA**, **Merck Sharp & Dohme Corp.** (Employee, Shareholder) **Jennifer A. Huntington, PharmD**, **Merck Sharp & Dohme Corp., a subsidiary of Merck & Co., Inc., Kenilworth, NJ, USA** (Employee) **Myra W. Popejoy, PharmD**, **Merck Sharp & Dohme Corp.** (Employee) **Mekki Bensaci, PhD**, **Merck Sharp & Dohme Corp., a subsidiary of Merck & Co., Inc., Kenilworth, NJ, USA** (Employee) **Carisa S. De Anda, PharmD**, **Merck Sharp & Dohme Corp.** (Employee, Shareholder) **Elizabeth G. Rhee, MD**, **Merck Sharp & Dohme Corp** (Employee, Shareholder) **Christopher Bruno, MD**, **Merck Sharp & Dohme Corp., a subsidiary of Merck & Co., Inc., Kenilworth, NJ, USA** (Employee)

